# Target-Specific Extracorporeal Shockwave Therapy Modulates TLR4/NF-κB/MMP13 Signaling Within the Joint Microenvironment to Improve Osteochondral Pathology in Patellofemoral Osteoarthritis

**DOI:** 10.7150/ijms.132904

**Published:** 2026-08-24

**Authors:** Shun-Wun Jhan, Kuan-Ting Wu, Wen-Yi Chou, Po-Cheng Chen, Wen-Chiung Huang, Jai-Hong Cheng

**Affiliations:** 1 Department of Orthopedic Surgery, Kaohsiung Chang Gung Memorial Hospital and Chang Gung University College of Medicine, Kaohsiung 833, Taiwan.; 2 Center for Shockwave Medicine and Tissue Engineering, Kaohsiung Chang Gung Memorial Hospital and Chang Gung University College of Medicine, Kaohsiung 833, Taiwan.; 3 Department of Physical Medicine and Rehabilitation, Kaohsiung Chang Gung Memorial Hospital, College of Medicine, Chang Gung University, Kaohsiung City 833, Taiwan.; 4 Medical Research, Kaohsiung Chang Gung Memorial Hospital and Chang Gung University College of Medicine, Kaohsiung 833, Taiwan.

**Keywords:** patellofemoral joint osteoarthritis, extracorporeal shockwave therapy, patellar bone, articular cartilage, inflammatory mediator

## Abstract

**Background:**

Patellofemoral joint (PFJ) osteoarthritis (OA) is characterized by cartilage degeneration, subchondral bone remodeling, fat pad fibrosis, and chronic synovial inflammation. This study investigated the therapeutic effects of extracorporeal shockwave therapy (ESWT) applied to different anatomical targets in a rat PFJ OA model.

**Methods:**

PFJ OA was induced in rats and treated with ESWT targeted to either the tibial side (SWT) or femoral side (SWF). Micro-computed tomography, histological staining, and immunohistochemistry were used to assess structural changes, fibrosis, and inflammation. Molecular assessments showed the direct and indirecd effects of ESWT on the key inflammatory mediators, including IL-1β, NF-κB, RAGE, TLR4, and MyD88, and significantly reduced MMP13 expression in joint tissues.

**Results:**

ESWT significantly attenuated osteophyte formation, cystic changes, and sclerosis in the femoral and patellar bone. SWT showed superior improvement in bone volume fraction, trabecular thickness, and patellar bone integrity. Histologically, ESWT reduced cartilage degradation, fat pad fibrosis, and synovial inflammation. Molecular analyses revealed downregulation of pro-inflammatory mediators and MMP13, with SWT producing greater effects than SWF.

**Conclusions:**

ESWT effectively mitigates structural damage, inflammation, and catabolic signaling in PFJ OA. Targeting the tibial side enhances therapeutic outcomes, highlighting ESWT as a promising non-invasive treatment strategy for PFJ OA.

## Introduction

Osteoarthritis (OA) of the knee is a common disorder that causes pain, stiffness and functional disability in daily activities. The knee joint is a tri-compartmental joint, which including patellofemoral (PF) joint, medial and lateral tibiofemoral (TF) joint. The PF joint is one of the most commonly affected compartments in knee joint. Recent study has observed a higher frequency of radiographic osteophytes in the PF joint compared with the TF joint compartment 1. Another study discovers most common radiographic pattern in patients with knees pain is combined TF joint and PF joint disease, followed by isolated PF joint osteoarthritis 2. These studies emphasize the important role of PF joint in knee osteoarthritis.

The pathogenesis of osteoarthritis includes systemic and local factors. The PF joint reaction force, measuring the compression of the patella against the femur, is one of the local factors. The distribution of PF joint reaction force associates with the alignment and motion of the patella within the femoral trochlea. Patella lateral tilting leads to increased contact pressure on the lateral facet and then cause PF joint osteoarthritis.

Extracorporeal shockwave therapy (ESWT) has chondroprotective effect in prevention and regression of osteoarthritis in joints in animal models. ESWT could improve the subchondral bone and had positive effect in treatment of patella lateral tilting related PF joint osteoarthritis.

Malalignment or tilt of patella causes patellofemoral lateral facet arthritis. Malalignment of extensor mechanism also results in overload on lateral aspect of the knee and patella subluxation. Kalichman et al. reports that patellar alignment, including patellar length ratio, sulcus angle, lateral tilt, and bisect offset, is significantly associated with features of patellofemoral osteoarthritis as seen on MRI, such as decreased cartilage thickness and the presence of bone marrow lesions 3. The treatment for PF joint osteoarthritis includes conservative treatment, soft tissue realignment, tibial tubercle osteotomy, autologous chondrocyte implantation, patellectomy, or joint replacement.

Wang et al. had reports application of ESWT to the subchondral bone of the medial tibia condyle shows regression of osteoarthritis of the knees in rats 4. Another study mentions that ESWT has treatment related chondroproctective effect in osteoarthritis of the knee in rats 5. Wang et al. also reports ESWT causes molecular changes that are consistent with improvement in subchondral bone remodeling and chondroprotective effect 6. In summary, ESWT exerts a protective effect on articular cartilage by promoting subchondral bone remodeling during the early development of osteoarthritis in an anterior cruciate ligament transection rat mode 6.

Previous studies focuse on ESWT on TF joint osteoarthritis of the knees to reduce inflammation and extracellular matrix proteins 7. Despite increasing evidence supporting the therapeutic effects of ESWT in knee osteoarthritis, most previous studies have predominantly focused on the tibiofemoral joint, with limited attention given to the patellofemoral joint, particularly in the context of patellar malalignment induced OA 8, 9. Furthermore, the optimal anatomical target for ESWT application in PFJ OA remains unclear, and whether site-specific delivery results in differential therapeutic outcomes has not been systematically investigated. In addition to structural degeneration, osteoarthritis is now widely recognized as a disease involving chronic low-grade inflammation and dysregulation of innate immune signaling. Key inflammatory mediators, including interleukin-1 beta (IL-1β), nuclear factor kappa B (NF-κB), receptor for advanced glycation end-products (RAGE), and toll-like receptor 4 (TLR4)/myeloid differentiation primary response 88 (MyD88) signaling pathways, are known to play critical roles in driving synovial inflammation, infrapatellar fat pad activation, and cartilage degradation through downstream effectors such as matrix metalloproteinase 13 (MMP13) 10-12. However, it remains unclear whether ESWT can modulate these inflammatory pathways in PFJ OA, and whether such effects are dependent on the anatomical site of treatment. Therefore, the aim of this study is to investigate the therapeutic effects of site-specific ESWT in a rat model of PFJ OA induced by patellar lateral tilting, with a particular focus on both structural outcomes and the modulation of key inflammatory signaling pathways.

## Materials and Methods

### Animals

Total thirty-two Sprague-Dawley rats (BioLasco, Taiwan) were randomly used in the experiment. The IACUC protocol of the animal study was approved by the Animal Care Committee of hospital. We have included an ARRIVE checklist to show that we have conformed to the ARRIVE guidelines. The animals were cared at the Center for laboratory Animals for 1 week before experiment. They were housed at 23 ± 1 °C with a 12-hour light and dark cycle and veterinarians took care of them for healthy, food and water.

### Patellofemoral joint osteoarthritis of rat knees model

The rats underwent anesthesia with Zoletil 50 (25 mg/Kg) and Xylazine (10 mg/Kg) for surgery on the left knee with sterile fashion. The plication of lateral retinaculum of knees was performed to induce patella lateral tilting, which lead to PF joint OA. Two sutures were performed over upper and lateral parts of lateral retinaculum of knees. The wound of surgery on the knee joint was irrigated and was closed. Prophylactic antibiotic with ampicillin (20 mg/Kg) and Ketorolac (1 mg/Kg/day) was given for 3 days after surgery. Post-surgery, the animals were returned to the housing cage and were taken care of by a veterinarian. The surgical site and the animal activities were observed daily. PF joint osteoarthritis was defined as wear of articular cartilage, joint space narrowing, bony spur and osteophyte formation, and joint deformity.

### Study design

In accordance with the principles of the 3Rs (Replacement, Reduction, and Refinement), we calculated the total number of rats required for each group based on the sample size determined using G*Power (version 3.1.9.7). The analysis indicated that eight rats per group would provide sufficient statistical power (0.8) to detect a 10 % difference in the experiment, using a previous study as a reference 13. In the experiment, 32 Sprague-Dawley (SD) rats were used in this study. The experimental procedure and timeline were shown in Figure 1A. All rats were randomly divided into 4 groups (8 rats for each group): Sham group was sham surgery and without treatment. Patellofemoral joint (PFJ) group was the rats that were received lateral retinaculum plication on the left knee without receiving treatment to create PF knee OA (Figure 1B). SWF group was the rats that were received lateral retinaculum plication on the left knee to create PF joint OA. One week after surgery, the rats were applied shock waves (SW) (0.25 mJ/mm^2^ with 800 impulses, 4 Hz) on the distal lateral femur of left knee (Figure 1C and 1D). SWT group was the rats that were received lateral retinaculum plication on the left knee to create PF joint OA. One week after surgery, the rats were applied SW (0.25 mJ/mm^2^ with 800 impulses, 4 Hz) on the proximal medial tibia of left knee (Figure 1C and 1E). All animals were scarified post-OA at 9 weeks for analysis. The macroscopic observation, histological analysis by hematoxylin and eosin (HE) stain, Safranin-O stain, synovitis score and micro-CT were used to observe the response of PF joint osteoarthritis to ESWT. Prevention of articular cartilage wear, subchondral bone remodeling, and decreasing synovitis score were measured the ESWT effects on PF joint osteoarthritis.

### Extracorporeal shockwave therapy

The animals with PF knee OA were treated with SW as SWF and SWT group (Figure 1C). The source of SW was generated using a DUOLITH SD1 (STORZ MEDICAL AG, Swiss) device. In SWF group, the SW was applied on the distal lateral femur condyle of the left knee at 0.5 cm up the joint line and 0.5 cm from the medial skin surface. In SWT group, the SW was applied on the medial tibia condyle of the left knee at 0.5 cm below the joint line and 0.5 cm from the medial skin surface. Each left knee received ESWT with 800 impulses at 4 Hz and an energy flux density of 0.25 mJ/mm². The treatment parameters were selected according to previous studies investigating the chondroprotective effects of ESWT in rat osteoarthritis models 14.

### Histopathological examination and OARSI score

The harvested joint specimens were fixed at 4 % PBS buffered formaldehyde at 4 °C for 48 hours and were decalcified in 10 % PBS-buffered EDTA solution at 25 °C for one month. Decalcified tissues were embedded in paraffin wax. The specimens were cut longitudinally into 5μm thick sections and transferred to poly-lysine-coated slides (Thermo Fisher Scientific, USA). The samples were stained with haematoxylin-eosin (HE) technique, and Safranin O (Sigma-Aldrich, St. Louis, MO, USA), and the articular cartilage of knees were graded histologically using Osteoarthritis Research Society International (OARSI) scores for assessments of pathological cartilage structure.

### Micro-CT scan

The harvested left knee specimens were subjected to micro-CT scan (SkyScan, 1076, Kartuizersweg 3B 2550 Kontich, Belgium) analysis. The left knee was prepared and sized to fit the micro-CT for scanning. The bone volume, bone surface, bone surface/volume ratio, bone surface density, trabecular thickness, trabecular number and bone porosity were measured and computer analyzed.

### Immunohistochemical analysis

The articular cartilage of patellofemoral region, retro-patellar fat pad and patellofemoral joint were analyzed with immunohistochemical analysis of anti-rat interleukin-1 beta (IL-1β) (1:150, PA5-119220, Thermo Fisher Scientific Inc., USA), nuclear factor kB p65 (NF-κB p65) (1:500, PA5-16545, Thermo Fisher Scientific Inc., USA), the Receptor for Advanced Glycation End-products (RAGE) (1:200, PA1-075, Thermo Fisher Scientific Inc., USA), toll-like receptor 4 (TLR4) (1: 100, MA5-16216, Thermo Fisher Scientific Inc., USA), myeloid differentiation primary response 88 (MyD88) (1:50, sc-74532, Santa Cruz Biotechnology Inc., USA) and matrix metallopeptidase 13 (MMP13) (1:500, PA5-95486, Thermo Fisher Scientific Inc., USA). The immuno-reactivity in samples was used HRP-DAB Cell & Tissue Staining Kit (R & D Systems, USA). The activities were quantified from five areas in three sections of the same specimen using a Zeiss Axioskop II plus microscope (Carl Zeiss, Germany). The images were collected by a Cool CCD camera (SNAP-Pro c.f. Digital kit; Media Cybernetics, USA). Images were analyzed by an Image-Pro® Plus software (Media Cybernetics, USA).

### Statistical analysis

Statistical analyses were conducted using SPSS version 17.0 (SPSS Inc., Chicago, IL, USA). All results presented as mean ± standard deviation (SD). The distribution of each variable was assessed using the Kolmogorov-Smirnov test to determine normality. For data following a normal distribution, comparisons were made using the paired Student's t-test. For non-normally distributed data, the Wilcoxon Signed-Rank test was applied for within-group comparisons, while the Mann-Whitney U test was used for between-group analyses. A P-value less than 0.05 was considered statistical significance.

## Results

### ESWT protected the bone damage in the PF joint of OA rat model

The micro-CT images revealed the different positions of ESWT application (femur condyle and tibia condyle) as well as the morphology of the femur medial subchondral bone and patellar bone after treatment (Figure 2A and 2B). The femur medial subchondral bone of PFJ group displayed cyst and osteophyte on the weight-bearing areas of the subchondral plate (Figure 2A). The Sham, SWF and SWT groups revealed no obvious erosion, trabecular bone thickening or sclerosis in the subchondral bone. The patellar bone of PFJ group exhibited severe superficial sclerosis and osteophyte formation as compared with Sham, SWF and SWT groups (Figure 2B).

The micro-CT data of subchondral bone of femur and patella bone were showing that SWF group significantly improved the bone volume (BV/TV) (P < 0.05) and trabecular thickness (Tb. Th.) (P < 0.05) (Figure 2C, 2D and 2E). The patella bone protective effect of SWT group (P < 0.001) was better than SWF group (P < 0.01) in BV/TV and Tb.Th. but no difference in Tb. N by compared with PFJ group (Figure 2F, 2G and 2H).

### Pathological analysis of PF joint OA after ESWT

Pathological changes of articular cartilage of tibia, infrapatellar fat pad and PF joint were analyzed by HE staining (Figure 3). The results of the tibial articular cartilage revealed severe cartilage degradation in the PFJ group, characterized by surface fibrillation, matrix loss, and chondrocyte clustering (Figure 3A). In contrast, the SWF and SWT groups showed relatively preserved cartilage structure with reduced matrix loss and chondrocyte disorganization. The SWT group exhibited the most evident improvement in cartilage integrity, approaching the histological appearance compared to the PFJ group (P < 0.05) (Figure 3A, right panel).

In addition, fibrotic changes in the infrapatellar fat pad were examined histologically (Figure 3B). The PFJ group demonstrated marked fibrosis, including increased extracellular matrix deposition, fibrous tissue expansion, and vascular proliferation (black arrows). These fibrotic features were attenuated in the SWF and SWT groups, with the SWT group showing the most notable reduction in fibrotic area and vascular density (P < 0.05) (Figure 3B, right panel). Quantitative analysis of fibrosis scores indicated a significant therapeutic effect of ESWT, particularly in the SWT group (P < 0.05 vs. PFJ) (Figure 3B, right panel).

Inflammatory cell infiltration in the PF joint space and synovium was assessed (Figure 3C). The PFJ group exhibited extensive inflammatory infiltration, synovial hyperplasia, and pannus formation (red arrows). Both SWF and SWT groups treatments markedly reduced synovial inflammation and pannus invasion. Among them, SWT group provided the most substantial reduction in inflammation (P < 0.05) (Figure 3C, right panel). Inflammatory scores were significantly decreased in both treatment groups compared to PFJ group, with the SWT group showing the lowest levels of inflammation (P < 0.05) (Figure 3C, right panel).

These results demonstrate that ESWT, particularly when applied to the tibia (SWT group), ameliorates key pathological features of PF joint OA, including cartilage degeneration, fat pad fibrosis, and joint inflammation.

### ESWT effects on IL-1β, NF-κB, and RAGE expression in joint tissues of PFJ OA

The IL-1β, NF-κB, and RAGE pathway is an important inflammatory signaling cascade. The expression of IL-1β, NF-κB and RAGE were measured in joint tissues of PF joint OA (Figure 4 and Table 1). Compared with the Sham group, PF joint OA markedly increased IL-1β expression in the articular cartilage (P < 0.001) while reducing its levels in the infrapatellar fat pad and PF joint (both P < 0.001). Both SWF and SWT groups treatments significantly reduced IL-1β in articular cartilage (P < 0.05 as compared with PFJ group), infrapatellar fat pad (both P < 0.001) and PF joint (both P < 0.001).

For NF-κB, PFJ OA significantly elevated expression in all tissues compared with Sham group (all P < 0.001). In the infrapatellar fat pad and PF joint, both SWF and SWT groups reduced NF-κB levels compared to PFJ group (P < 0.01 or P < 0.05), with SWT group showing greater reductions in the fat pad (P < 0.001 as compared with PFJ group; P < 0.001 as compared with SWF group) but without significant in PF joint (P > 0.05) (Table 1). However, in articular cartilage, neither treatment significantly lowered NF-κB expression relative to PFJ group.

Regarding RAGE, PF joint OA led to substantial increases in all three joint regions (all P < 0.001 as compared with Sham). SWT group significantly reduced RAGE expression in the infrapatellar fat pad (P < 0.05 as compared with PFJ group; P < 0.01 as compared with SWF group), while neither SWF group nor SWT group achieved significant reductions in articular cartilage or patellofemoral joint compared to PFJ group.

### ESWT mitigated TLR4/MyD88-mediated inflammation and MMP13-driven cartilage degradation in PF joint OA

Compared to the Sham group, PF joint OA significantly increased TLR4 and MyD88 expression in the articular cartilage, infrapatellar fat pad, and PF joint (all P < 0.05) (Figure 5 and Table 1). Both treatments of SWF and SWT groups reduced TLR4 expression in the PF joint (P < 0.001 as compared with PFJ group), while only SWT group decreased TLR4 levels in the infrapatellar fat pad (P < 0.001). In articular cartilage, neither SWF group nor SWT group showed significant reductions in TLR4 expression compared to PFJ group. For MyD88, only SWT group additionally led to reduction and better than SWF group in infrapatellar fat pad and PF joint of MyD88 levels (P < 0.01 and P < 0.05, SWF group as compared with SWT group).

Concerning MMP13, PFJ OA markedly elevated its expression in the infrapatellar fat pad and PF joint (P < 0.001 as compared with Sham). Both SWF and SWT groups reduced MMP13 in the infrapatellar fat pad, with SWT group showing a stronger effect (P < 0.001 as compared with PFJ group; SWF as compared with SWT group, P < 0.05). In the PF joint, SWT group significantly decreased MMP13 expression compared to PFJ group (P < 0.01), while SWF group did not show significant differences. No significant changes in articular cartilage MMP13 expression were observed among groups.

## Discussion

Our study demonstrated that ESWT effectively mitigates critical pathological changes associated with PF joint OA in a rat model. Micro-CT analysis revealed that PF joint OA was characterized by subchondral bone cyst formation, osteophyte development, and sclerosis, consistent with clinical observations of advanced OA progression. Importantly, both SWF group and SWT group regimens preserved trabecular bone microarchitecture, increasing BV/TV, trabecular thickness, and number. The SWT group exhibited superior bone-protective effects in the patella, suggesting that precise localization of shock wave delivery enhances therapeutic efficacy. Pathologically, ESWT improved articular cartilage integrity, reduced matrix loss, and alleviated chondrocyte clustering. Moreover, it substantially attenuated infrapatellar fat pad fibrosis and joint-space inflammatory infiltration. The reduction in pannus formation and synovial hyperplasia highlights capacity of ESWT to modulate not only structural but also inflammatory components of OA pathology. These findings position ESWT as a promising non-invasive intervention capable of modifying disease progression by preserving joint structure and reducing inflammation, supporting its potential translation into clinical treatment strategies for early or moderate PF joint OA.

Beyond gross morphological preservation, our results revealed that ESWT exerts significant anti-inflammatory effects at the molecular level within joint tissues. Analysis of IL-1β, NF-κB, and RAGE expression demonstrated that PF joint OA robustly upregulated these pro-inflammatory mediators across articular cartilage, infrapatellar fat pad, and the PF joint. It has been reported that IL-1β, NF-κB, and RAGE are the key factors in the inflammation and pathogenesis of OA disease 10. Notably, SWT and SWF groups application significantly reduced IL-1β levels in the articular cartilage, infrapatellar fat pad and PF joint (Figure 4 and Table 1). IL-1β is known to promote the secretion of various inflammatory cytokines, adipokines, and growth factors from the infrapatellar fat pad, such as tumor necrosis factor alpha (TNF-α), IL-6, and vascular endothelial growth factor (VEGF) 15. This result suggests that SWT and SWF groups may better target deep periarticular tissues, effectively dampening inflammatory cascades. Similarly, both SWF and SWT groups reduced NF-κB activation in the fat pad and PF joint, with SWT group yielding greater suppression in the fat pad. Given central role of NF-κB in OA-associated synovial inflammation and cartilage catabolism, these results imply that ESWT may halt the amplification of inflammatory signaling networks 16. Furthermore, SWT group significantly reduced RAGE expression in the fat pad, underscoring its ability to interfere with advanced glycation end-product signaling and its contribution to chronic joint inflammation. Protein levels of the RAGE and its ligands, including advanced glycation end products (AGEs) and high-mobility group box-1 (HMGB-1), have been found to be elevated in knee joint tissues of OA knee patients with metabolic syndrome compared to those without metabolic syndrome 17. Together, these findings indicate that ESWT, particularly SWT group, can modulate key inflammatory pathways in OA progression, offering a mechanistic rationale for its protective effects.

The TLR4/MyD88 pathway is a well-established upstream regulator of NF-κB-mediated inflammatory and catabolic responses in osteoarthritis. Activation of TLR4 by damage-associated molecular patterns (DAMPs) leads to recruitment of MyD88 and subsequent activation of NF-κB signaling, which in turn induces the transcription of pro-inflammatory cytokines and matrix-degrading enzymes, including MMP13 18. Importantly, experimental studies have demonstrated that activation of TLR4 signaling directly increases MMP13 expression in chondrocytes, whereas pharmacological inhibition of TLR4 significantly suppresses both NF-κB activation and downstream catabolic mediators such as MMP13 19. In addition, inhibition of upstream TLR signaling has been shown to reduce NF-κB activation and concomitantly decrease MMP13 expression in osteoarthritic cartilage, further supporting a causal relationship between these pathways 20. Moreover, recent mechanistic studies indicate that MyD88-dependent signaling is required for the production of matrix metalloproteinases (MMPs) in osteoarthritic chondrocytes, linking innate immune activation directly to cartilage catabolism. Collectively, these findings support a signaling cascade in which TLR4/MyD88 activation drives NF-κB dependent transcription of MMP13, thereby promoting cartilage matrix degradation 21. ESWT exerts its biological effects primarily through mechanotransduction, converting mechanical stimuli into intracellular signaling. This process activates pathways such as MAPK and PI3K/Akt, and has been shown to suppress NF-κB signaling, a key regulator of inflammatory cytokines in osteoarthritis 22, 23. In our study, ESWT may attenuate innate immune responses by modulating TLR4/MyD88 signaling, thereby reducing downstream NF-κB activation and catabolic enzymes such as MMP13. These findings are consistent with previous studies demonstrating that activation of the TLR4/MyD88 pathway promotes NF-κB-dependent transcription of MMP13, whereas inhibition of TLR4 signaling suppresses both NF-κB activation and downstream catabolic mediators in osteoarthritic cartilage. Recent studies have emphasized that osteoarthritis is a microenvironment-driven disease involving complex interactions among inflammation, oxidative stress, and aging, and that effective therapies should be capable of modulating these local pathological processes in a spatially targeted manner 24-26. In this regard, the ability of ESWT to regulate inflammatory signaling pathways may reflect a form of mechanobiological intervention that reshapes the joint microenvironment.

The study further demonstrated that ESWT modulates the TLR4/MyD88 expression and downstream cartilage-degrading enzyme MMP13, both of which are critical mediators in OA pathogenesis (Figure 5). PFJ OA was associated with significantly elevated TLR4 and MyD88 expression in all joint regions, reflecting heightened innate immune activation and inflammatory signaling 27-29. SWT group application notably reduced TLR4 expression in the infrapatellar fat pad and PF joint, while SWF group was less effective, suggesting that targeting tibial subchondral structures may more effectively intercept danger-associated molecular pattern (DAMP)-driven inflammation 29. Only SWT group reduced MyD88 expression to achieving greater suppression in the fat pad and PF joint. This differential effect implies that shock wave parameters and delivery sites can be optimized to achieve targeted molecular modulation. Moreover, MMP13 expression, markedly elevated in the infrapatellar fat pad of PFJ OA rats, was significantly reduced by both ESWT modalities, with SWT group showing a superior inhibitory effect. The role of MMP13 in collagen matrix degradation and OA cartilage erosion underscores the relevance of this finding 30. Collectively, these results highlight the capacity of ESWT to interfere with innate immune signaling and catabolic enzyme production, which are key drivers of OA progression.

These findings highlight the therapeutic potential of ESWT as a non-invasive, tissue-preserving intervention for PFJ OA, addressing both structural damage and underlying molecular pathology. The observed superiority of SWT group over SWF group in several parameters suggests that the site and energy parameters of shock wave delivery are critical determinants of clinical efficacy. This has direct implications for human application, where targeting subchondral bone or periarticular structures may yield differential outcomes. In addition, the differential molecular responses between periarticular soft tissues and articular cartilage may be related to both the characteristics of the experimental model and the intrinsic biological properties of these tissues 31, 32. In this study, osteoarthritis-like changes were induced using a patellar eversion procedure that primarily affects the PF joint and surrounding periarticular tissues rather than directly destabilizing the TF joint (Figure 3). As a result, inflammatory activation is more likely to occur in tissues such as the intrapatellar fat pad and synovium, while articular cartilage experiences relatively limited primary injury. In addition, periarticular tissues are highly vascularized and metabolically active, making them more responsive to inflammatory stimuli and therapeutic interventions 33. In contrast, articular cartilage is avascular and has low cellular density, which may limit rapid molecular responses. Furthermore, ESWT primarily acts through mechanotransduction pathways that promote angiogenesis and anti-inflammatory signaling, processes that are more prominent in vascularized periarticular tissues than in cartilage 34.

Although the lateral femoral condyle is the primary site of abnormal mechanical loading in this model, our findings suggest that the therapeutic effects of ESWT may extend beyond the site of maximal stress. Recent evidence suggests that osteoarthritis is a whole-joint disease involving extensive communication among subchondral bone, synovium, infrapatellar fat pad, and cartilage 35. ESWT applied to the proximal medial tibia may influence the entire joint microenvironment through subchondral bone remodeling and mechanobiological signaling. Since the SWT group showed the greatest suppression of inflammatory mediators within the infrapatellar fat pad, it is possible that modulation of IFP-derived inflammatory signaling contributed to the superior structural outcomes observed (Table 1). Therefore, the benefits of SWT may not solely depend on proximity to the region of maximal mechanical stress, but rather on its ability to induce favorable mechanotransductive responses across interconnected joint tissues.

However, several limitations warrant consideration. The study was performed in a rat model, which, while relevant, may not fully present the complexity of human OA pathology and biomechanics. Moreover, long-term durability of ESWT effects remains unclear, necessitating further studies with extended follow-up. Future research should explore optimized dosing regimens, combinatorial therapies with biologics or anti-inflammatories, precise imaging-guided delivery techniques and behavioral assessments such as gait analysis or weight-bearing distribution to correlate tissue changes with functional improvement. Additionally, elucidating the detailed mechanotransductive mechanisms underlying the effect of ESWT will be critical to refine its clinical use. Despite these challenges, our findings provide strong preclinical evidence supporting ESWT as a promising strategy to modify OA disease course, meriting further translational and clinical investigation. The lateral retinaculum plication model used in this study primarily represents a mechanically induced form of osteoarthritis characterized by patellofemoral malalignment and altered joint loading. While this model effectively reproduces structural degeneration, it does not fully encompass the complex, low-grade systemic inflammation observed in age-related or metabolic osteoarthritis. Nevertheless, accumulating evidence indicates that mechanical stress can directly activate innate immune signaling pathways, including TLR4/NF-κB, thereby linking biomechanical dysfunction to inflammatory responses 18, 27. In addition, the infrapatellar fat pad has been shown to exhibit an inflammatory phenotype in osteoarthritis, contributing to local cytokine production and disease progression 31. Consistent with these findings, our results demonstrated increased expression of IL-1β, NF-κB, and MMP13 in joint tissues, suggesting that this model partially recapitulates inflammation-driven mechanisms. However, caution is warranted when extrapolating these findings to human osteoarthritis, particularly in the context of aging or metabolic factors, which involve systemic and chronic inflammatory processes beyond localized mechanical stress.

The ESWT parameters used in this study (0.25 mJ/mm², 800 impulses, 4 Hz) were selected based on prior experimental studies demonstrating chondroprotective and subchondral bone modulating effects in osteoarthritis models 14. In particular, ESWT targeting subchondral bone has been reported to yield superior therapeutic outcomes compared to cartilage-targeted application, supporting the rationale for the parameter selection in this study.

Nevertheless, ESWT is known to exhibit dose-dependent biological effects, and variations in energy level, impulse number, frequency, and treatment timing may influence therapeutic efficacy 36. It is therefore possible that different parameter settings could yield different outcomes, particularly in the SWF group, which demonstrated relatively lower efficacy. In addition to anatomical targeting, tissue-specific factors such as vascularity, mechanical properties, and energy transmission depth may also affect the responsiveness to ESWT. Importantly, a single standardized protocol was intentionally applied across all treatment groups in this study to isolate the effect of treatment location and minimize confounding variables. Future studies are warranted to systematically investigate optimal ESWT dosing strategies, including repeated applications and parameter adjustments tailored to specific anatomical targets.

## Conclusion

This study demonstrates that ESWT effectively attenuates structural damage and inflammatory responses in a rat model of PFJ OA. ESWT preserved subchondral bone architecture, improved cartilage integrity, reduced fat pad fibrosis, and mitigated synovial inflammation. ESWT modulated inflammatory signaling predominantly within periarticular tissues, including the infrapatellar fat pad and PF joint, which may contribute indirectly to its chondroprotective effects. Notably, SWT group consistently achieved superior outcomes compared to femoral application (SWF group), highlighting the importance of treatment localization. These findings support ESWT as a promising, non-invasive therapeutic strategy to slow OA progression by targeting both structural and molecular disease mechanisms, warranting further translational research and clinical evaluation to optimize its application in human OA management.

## Figures and Tables

**Figure 1:**
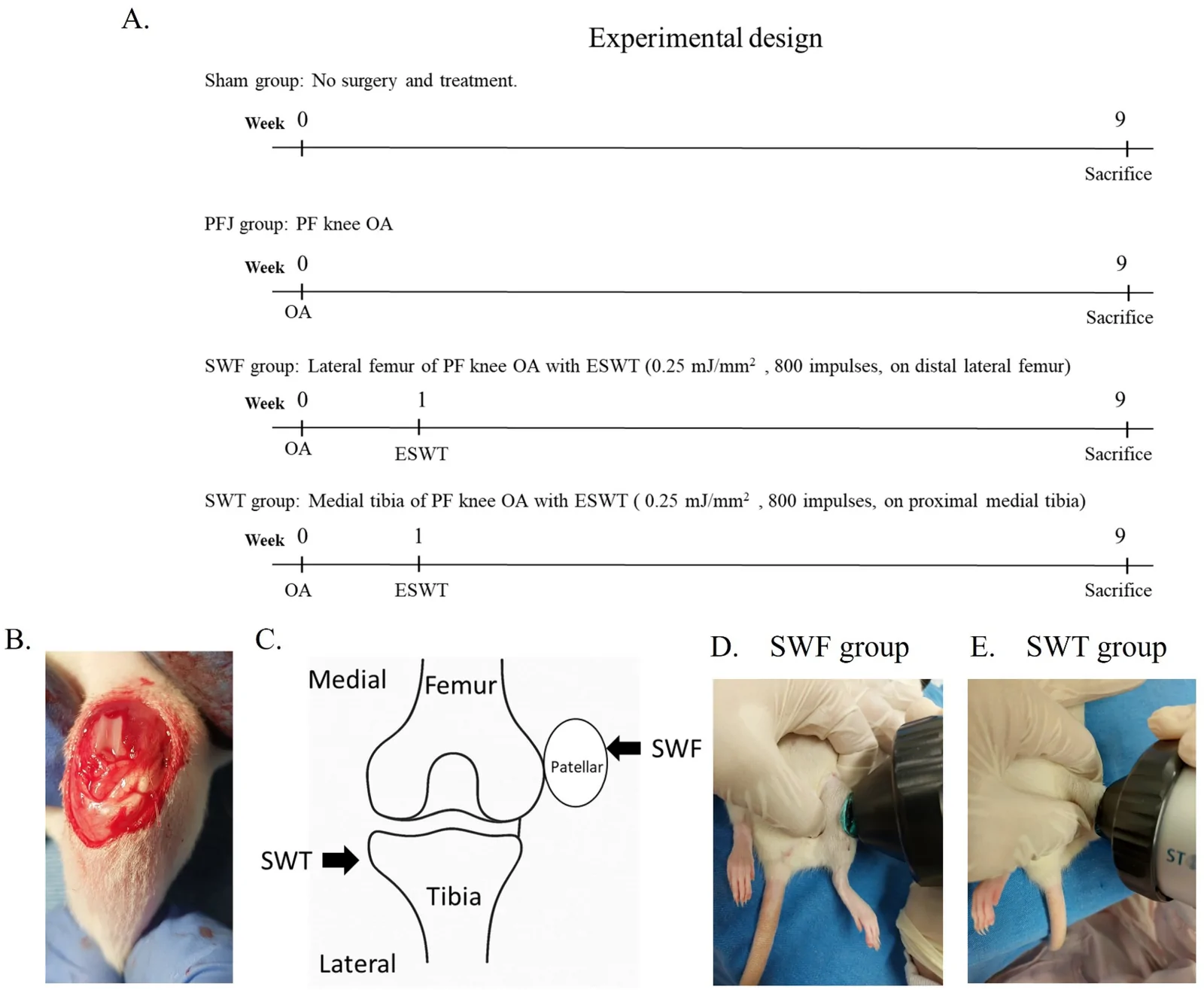
** (A)** The experimental design. **(B)** The surgery to create PF joint OA in left of rat knee. **(C)** The cartoon for application of ESWT on epiphyseal plate of left knee of rat. **(D)** The treatment of SWF goup on left of rat knee.** (E)** The treatment of SWT group on left of rat knee. N=8. ESWT = extracorporeal shockwave therapy.

**Figure 2:**
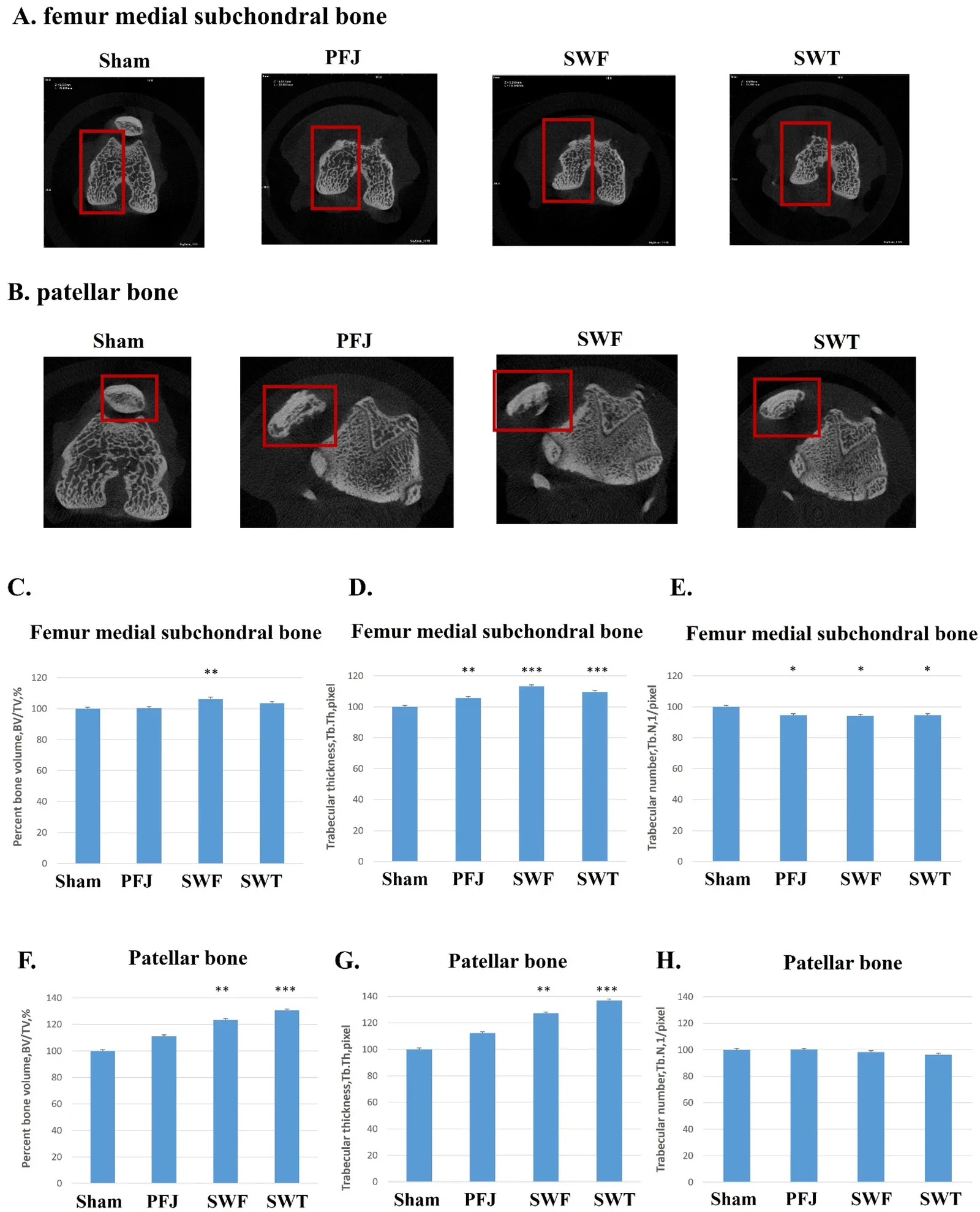
** Micro-CT images of femur medial subchondral bone and patellar bone. (A)** The photomicrographs of femur medial subchondral bone in transverse views from micro-CT. **(B)** the photomicrographs of patellar bone in transverse views. **(C)** The percentage of bone volume (BV/TV) in the region of interest of femur medial subchondral bone.** (D)** The percentage of trabecular thickness (Tb. Th) in the region of interest of femur medial subchondral bone. **(E)** The percentage of trabecular number (Tb. N) in the region of interest of femur medial subchondral bone. **(F)** The percentage of bone volume (BV/TV) in the region of interest of patellar bone. **(G)** The percentage of trabecular thickness (Tb. Th) in the region of interest of patellar bone. **(H)** The percentage of trabecular number (Tb. N) in the region of interest of patellar bone. The region of red line is the region of interest (ROI). N = 8 for each group. ^*^P < 0.05, 0.01 and 0.001 as compared with Sham group.

**Figure 3:**
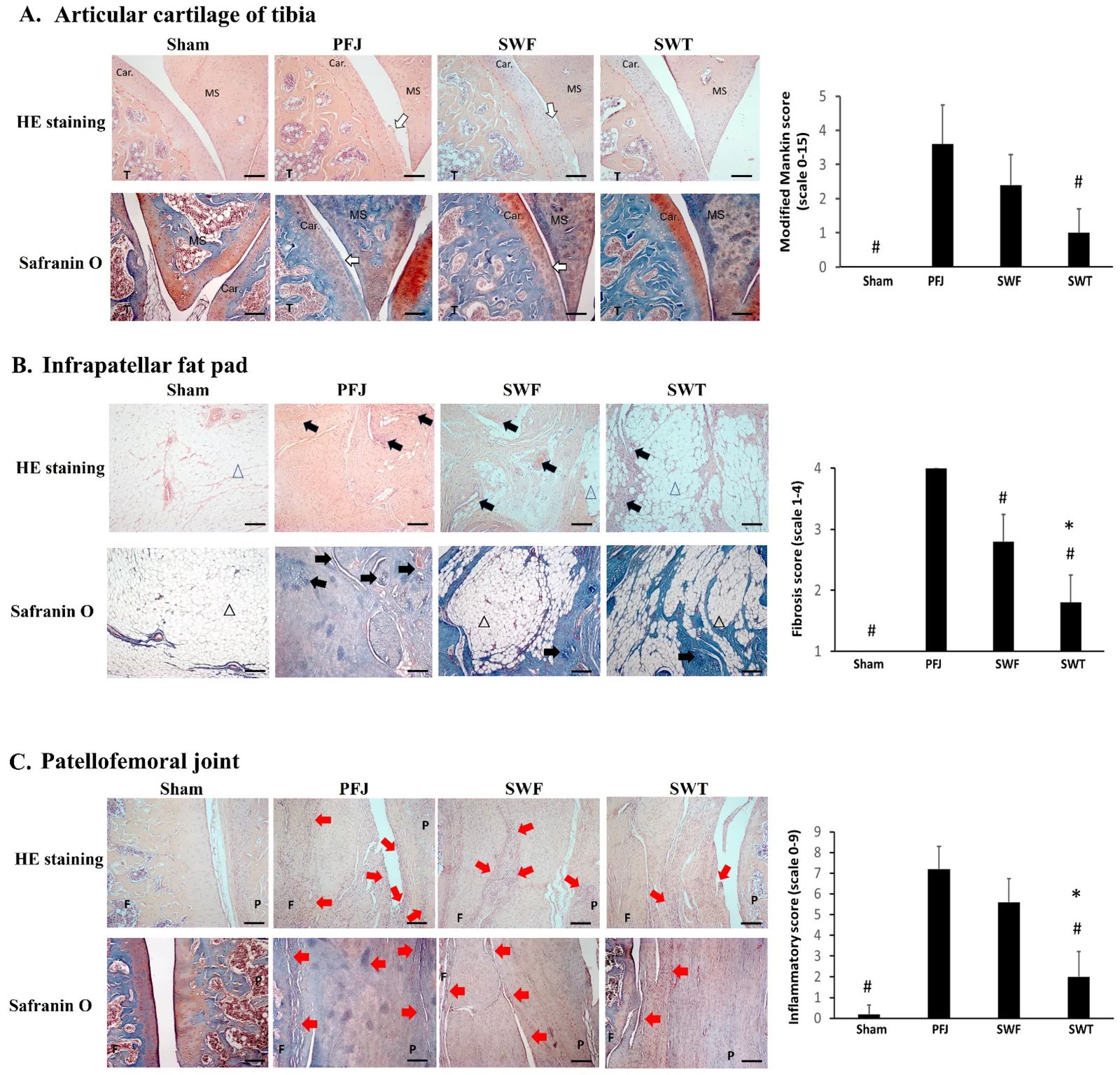
** Pathological analysis of articular cartilage of tibia, infrapatellar fat pad and PF joint in the patellofemoral joint (PFJ) osteoarthritis (OA) following ESWT. (A)** The modified Mankin score of articular cartilage of tibia. **(B)** Fibrosis score (scale 1-4) of infrapatellar fat pad. **(C)** Inflammatory score (scale 0-9) of PF joint. N = 8. Car.= Articular cartilage. MS = Meniscus. F = Femur. T = Tibia. P = patella. White arrow indicates rough cartilage surface by wearing. Black arrow indicates the fibrosis tissue. Triangle indicates the fat tissue. Red arrow indicates cell lining, resident cells and inflammatory infiltration. ^#^P < 0.05 as compared with PFJ group. ^*^P < 0.05 as SWT group compared with SWF group. The scale bar indicated 200 μm.

**Figure 4:**
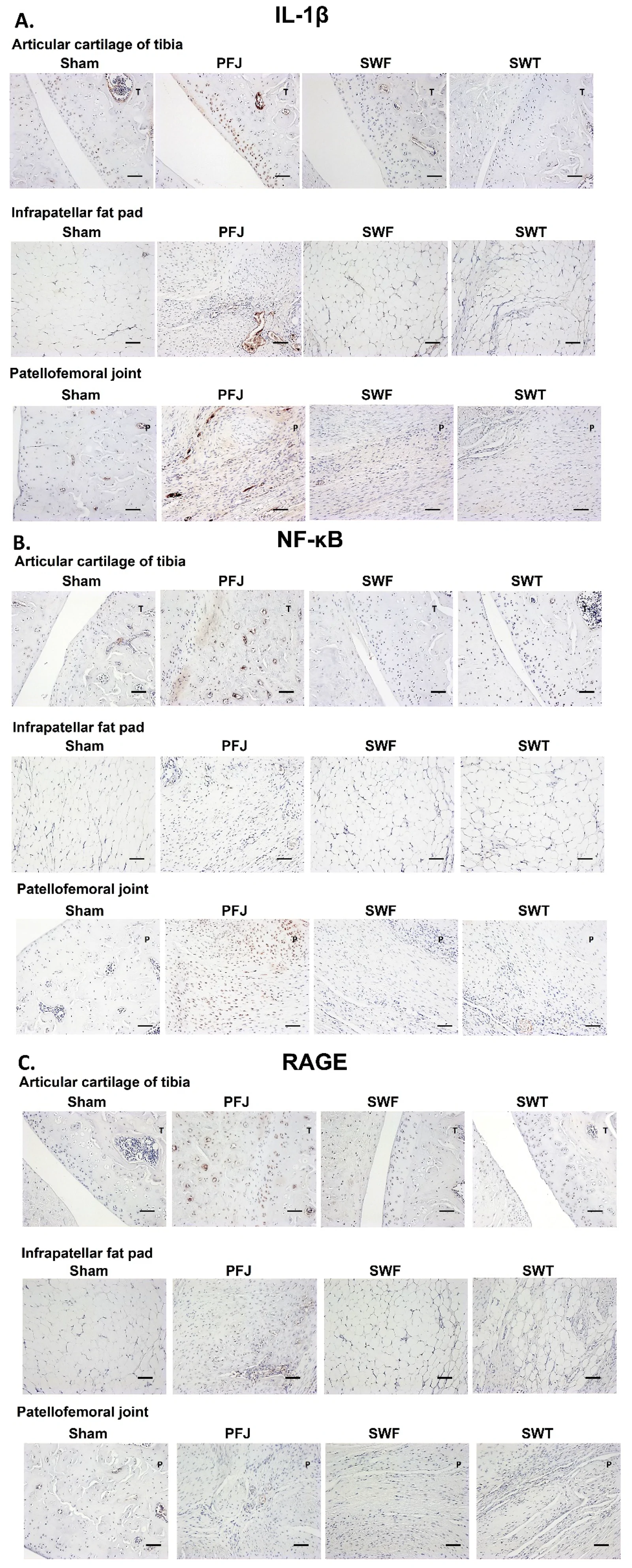
The immunohistochemistry stainings of** (A)** IL-1β, **(B)** NF-κB and** (C)** RAGE in articular cartilage of tibia, infrapatellar fat pad and PF joint in Sham, PFJ, SWF and SWT groups. The scale bar indicated 200 μm. N = 8.

**Figure 5:**
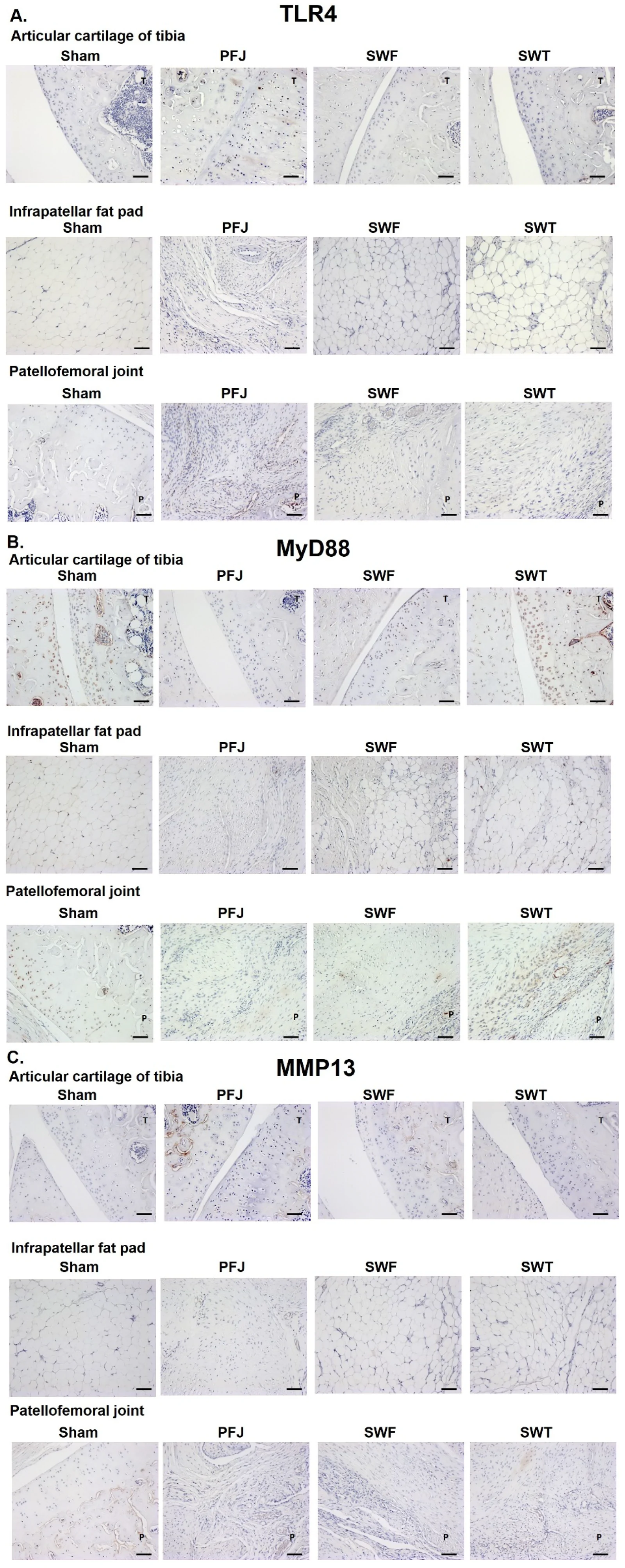
The immunohistochemistry stainings of **(A)** TLR4, **(B)** MyD88 and **(C)** MMP13 in articular cartilage of tibia, infrapatellar fat pad and PF joint in Sham, PFJ, SWF and SWT groups. The scale bar indicated 200 μm. N = 8.

**Table 1:** The expression of specific biomarkers after ESWT including IL-1β, NFkB, RAGE, TLR4, MYD88 and MMP13.

IL-1β	Articular cartilage	P-value*	P-value#	Infrapatellar fat pad	P-value*	P-value#	Patellofemoral joint	P-value*	P-value#
Sham	5.03±0.65	< 0.001		6.2±0.33	< 0.001		7.09±0.23	< 0.001	
PFJ	8.92±0.49			7.81±0.2			9.71±0.18		
SWF	6.53±0.77	< 0.05		5.23±0.16	< 0.001		6.79±0.31	< 0.001	
SWT	7.02±0.7	< 0.05	> 0.05	5.82±0.25	< 0.001	< 0.01	6.49±0.23	< 0.001	> 0.05
NF-κB	Articular cartilage	P-value*	P-value#	Infrapatellar fat pad	P-value*	P-value#	Patellofemoral joint	P-value*	P-value#
Sham	5.08±0.52	< 0.001		5.43±0.43	< 0.001		7.9±0.26	< 0.001	
PFJ	8.17±0.24			19.16±0.32			14.62±0.53		
SWF	7.23±0.35	> 0.05		16.64±0.57	< 0.01		13.15±0.33	< 0.05	
SWT	7.73±0.29	> 0.05	> 0.05	11.8±0.45	< 0.001	< 0.001	13.78±0.61	> 0.05	> 0.05
RAGE	Articular cartilage	P-value*	P-value#	Infrapatellar fat pad	P-value*	P-value#	Patellofemoral joint	P-value*	P-value#
Sham	4.51±0.31	< 0.001		3.55±0.34	< 0.001		6.16±0.36	< 0.001	
PFJ	7.49±0.21			14.68±0.78			13.81±0.29		
SWF	7.32±0.16	> 0.05		14.73±0.9	> 0.05		12.93±0.28	> 0.05	
SWT	7.23±0.46	> 0.05	> 0.05	10.96±0.56	< 0.05	< 0.01	12.67±0.19	< 0.01	> 0.05
TLR4	Articular cartilage	P-value*	P-value#	Infrapatellar fat pad	P-value*	P-value#	Patellofemoral joint	P-value*	P-value#
Sham	3.46±0.35	< 0.001		3.33±0.25	< 0.001		5.21±0.21	< 0.001	
PFJ	6.28±0.44			13.24±0.43			8.46±0.16		
SWF	5.95±0.3	> 0.05		13.51±0.4	> 0.05		7.19±0.81	< 0.001	
SWT	5.65±0.44	> 0.05	> 0.05	10.26±0.31	< 0.001	< 0.001	7.03±0.25	< 0.001	> 0.05
MyD88	Articular cartilage	P-value*	P-value#	Infrapatellar fat pad	P-value*	P-value#	Patellofemoral joint	P-value*	P-value#
Sham	8.67±0.23	< 0.05		9.98±0.21	< 0.001		10.15±0.23	< 0.01	
PFJ	9.13±0.35			15.99±0.34			11.58±0.3		
SWF	9.57±0.16	> 0.05		15.85±0.2	> 0.05		12.03±0.3	> 0.05	
SWT	9.96±0.36	> 0.05	> 0.05	14.61±0.24	< 0.01	< 0.01	10.60±0.51	< 0.05	< 0.05
MMP13	Articular cartilage	P-value*	P-value#	Infrapatellar fat pad	P-value*	P-value#	Patellofemoral joint	P-value*	P-value#
Sham	3.52±0.51	> 0.05		4.17±0.47	< 0.001		6.13±0.72	< 0.001	
PFJ	4.14±0.24			14.94±0.51			3.43±0.36		
SWF	4.05±0.17	> 0.05		11.24±0.74	< 0.01		3.25±0.36	> 0.05	
SWT	4.15±0.28	> 0.05	> 0.05	9.63±0.39	< 0.001	< 0.05	5.52±0.5	< 0.01	< 0.01

The ^*^p-value < 0.05 is as compared with PFJ groups. The ^#^p-value < 0.05 is as compared between SWF and SWT groups.

## Abbreviations

PFJ: patellofemoral joint
OA: osteoarthritis
ESWT: extracorporeal shockwave therapy
SWT: ESWT targeted to the tibial side
SWF: ESWT targeted to the femoral side
PF: patellofemoral
TF: tibiofemoral
SW: shock waves
HE: hematoxylin and eosin
OARSI: Osteoarthritis Research Society International
IL-1β: interleukin-1 beta
NF-κB p65: nuclear factor kB p65
RAGE: Receptor for Advanced Glycation End-products
TLR4: toll-like receptor 4
MyD88: myeloid differentiation primary response 88
MMP13: matrix metallopeptidase 13
BV/TV: bone volume
Tb. Th.: trabecular thickness
Tumor Necrosis Factor alpha: TNF-α
VEGF: vascular endothelial growth factor
AGEs: Advanced glycation end products
HMGB-1: high-mobility group box-1
DAMP: danger-associated molecular pattern
